# Primary Hyperparathyroidism Presenting as Posterior Reversible Encephalopathy Syndrome: A Report of Two Cases

**DOI:** 10.4274/jcrpe.galenos.2020.2019.0181

**Published:** 2020-11-25

**Authors:** Rimesh Pal, Aditya Dutta, Kanhaiya Agrawal, Nimisha Jain, Pinaki Dutta, Anil Bhansali, Arunanshu Behera, Sanjay Kumar Bhadada

**Affiliations:** 1 Post Graduate Institute of Medical Training and Research, Clinic of Endocrinology, Chandigarh, India; 2 Post Graduate Institute of Medical Training and Research, Clinic of General Surgery, Chandigarh, India

**Keywords:** Hypercalcemia, posterior reversible encephalopathy syndrome, primary hyperparathyroidism

## Abstract

Posterior reversible encephalopathy syndrome (PRES) is a clinico-radiological entity characterized by subcortical vasogenic edema presenting with acute neurological symptoms. Common precipitating causes include renal failure, pre-eclampsia/eclampsia, post-organ transplant, and cytotoxic drugs. Hypercalcemia is a rare cause of PRES; most cases occur in the setting of severe hypercalcemia secondary to malignancy or iatrogenic vitamin D/calcium overdose. Primary hyperparathyroidism (PHPT), as a cause of PRES, is an oddity. We report two cases of adolescent PHPT presenting with generalized tonic-clonic seizures and altered sensorium. On evaluation, both had hypertension, severe hypercalcemia (serum calcium 14.1 mg/dL and 14.5 mg/dL, respectively) and elevated parathyroid hormone levels. Magnetic resonance imaging (MRI) revealed T2/fluid-attenuated inversion recovery hyperintensities located predominantly in the parieto-occipital regions, suggestive of PRES. Identification and excision of parathyroid adenoma led to the restoration of normocalcemia. Neurological symptoms and MRI changes improved subsequently. An extensive literature search revealed only four cases of PHPTassociated PRES; none of them being in the pediatric/adolescent age group. The predominant clinical manifestations were seizures and altered sensorium. All had severe hypercalcemia; three had hypertension at presentation, while one was normotensive. Parathyroid adenomectomy led to normalization of serum calcium and resolution of neurological symptoms and radiological changes. Thus, severe hypercalcemia, although rare in PHPT, can lead to hypercalcemic crisis precipitating acute hypertension that can result in cerebral endothelial dysfunction with the breakdown of the blood-brain barrier, culminating in PRES. We therefore recommend that serum calcium levels should be checked in all patients with PRES and that PHPT be regarded as a differential diagnosis in those with underlying hypercalcemia.

**What is already known on this topic?**

Hypercalcemia, mostly severe hypercalcemia secondary to malignancies, has occasionally been implicated in the causation of posterior reversible encephalopathy syndrome (PRES). Primary hyperparathyroidism (PHPT) is usually associated with mild-moderate hypercalcemia and has rarely been implicated in PRES.

**What this study adds?**

Herein, we report two cases of adolescent PHPT presenting as PRES. We propose that serum calcium levels should be checked in all patients with PRES and that PHPT be regarded as a differential diagnosis in those with underlying hypercalcemia.

## Introduction

Posterior reversible encephalopathy syndrome (PRES) refers to a disorder of subcortical vasogenic edema in patients presenting with acute neurological symptoms, namely altered sensorium, seizures, headache, visual disturbances, and rarely, focal neurological deficits (1). Radiologically, it is characterized by the presence of bilateral hemispheric edema predominantly involving, but not solely restricted to, the parieto-occipital lobes. PRES has a good prognosis; the clinical and radiological features are reversible over days to weeks. The basic underlying pathophysiology is brain endothelial injury resulting from abrupt changes in blood pressure (BP) or direct toxic effects of cytokines on the endothelium that leads to the breakdown of the blood-brain barrier culminating in brain edema (1). Accordingly, PRES is well recognized in the settings of renal failure, preeclampsia/eclampsia, allogenic bone marrow transplantation, solid-organ transplantation, cytotoxic drugs, and autoimmune disorders (2). Hypercalcemia has rarely been implicated as a cause of PRES (3,4,5,6,7,8,9,10,11). However, all the reported cases had severe hypercalcemia, either secondary to malignancy (3,4,5,6,7,8), vitamin D toxicity (9), iatrogenic calcium infusion (10) or granulomatous infection (11). Primary hyperparathyroidism (PHPT) as a cause of PRES is an oddity with only a few cases reported in world literature (12,13,14,15). Herein, we report two cases of PHPT presenting as PRES and thereafter all the anecdotal cases hitherto reported in world literature are summarized.

## Case Report

### Case 1

**Figure 1:**
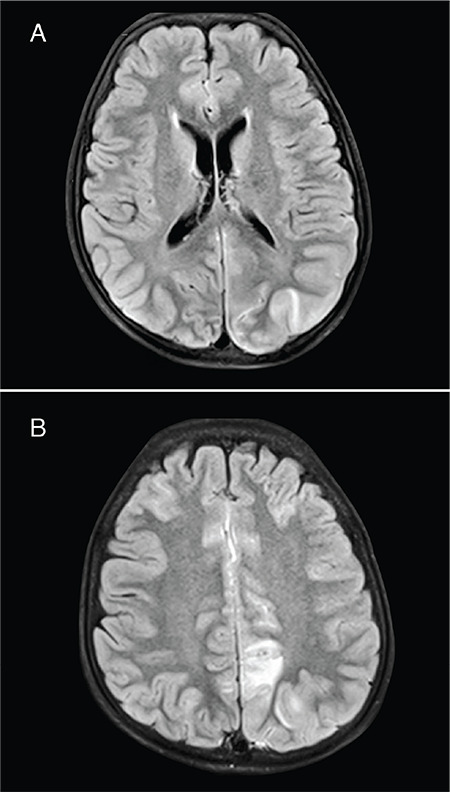
A, B) Magnetic resonance imaging of brain (first case) with T2/fluid-attenuated inversion recovery images showing hyperintensities in the parieto-occipital regions, predominantly on the left side

A 12-year-old boy presented with upper abdominal pain and recurrent episodes of vomiting for four days. The day before admission he had one episode of generalized tonic-clonic convulsion (GTCS) lasting for about 30 seconds that was followed by altered sensorium. At presentation to the emergency department (ED), he had impaired mentation, irrelevant talk, and a Glasgow Coma Scale (GCS) of 12/15 (E4V3M5). Pupils were bilaterally reacting to light. Vitals recorded were: pulse rate-96/min; BP-140/100 mmHg (mean arterial pressure-120 mmHg; >99th centile for age); respiratory rate 28/min; and capillary refill time <2 seconds. He had an upper abdominal tenderness; the rest of the physical examination was unremarkable. While at the ED, he had another episode of GTCS and was immediately started on phenytoin. BP control required labetalol infusion. Preliminary investigations revealed an elevated total leukocyte count of 18000/µL, increased serum calcium [corrected serum calcium 14.1 mg/dL (range: 8.8-10.4)], low serum phosphorous [2 mg/dL (range: 3.7-5.4)], and high serum amylase [1917 IU/L (range: 19-86)] and lipase [641 IU/L (range: 12-70)]. Serum creatinine was normal. The cerebrospinal fluid analysis was unremarkable. Ultrasonography of the abdomen showed a bulky pancreas with peri-pancreatic fat stranding (suggestive of pancreatitis) and bilateral nephrolithiasis. Non-contrast computerized tomography (CT) of the head was non-contributory; hence contrast-enhanced magnetic resonance imaging (CEMRI) was performed which was suggestive of T2/fluid-attenuated inversion recovery (T2/FLAIR) hyperintensities, with diffusion restriction involving the parieto-occipital areas (predominantly left-sided), indicative of PRES (Figure 1A, 1B). Hypercalcemia was managed with parenteral hydration and furosemide. Thereafter he was moved to the pediatric intensive care unit (ICU). Labetalol infusion, phenytoin, and parenteral fluids were continued. On day 3, his serum calcium came down to 11.8 mg/dL and BP was under control. His sensorium improved, however, he remained somewhat drowsy. Detailed work-up at ICU showed elevated serum intact parathyroid hormone (iPTH) [iPTH 203 pg/mL (range: 15-65)] and 25-hydroxyvitamin D of 22.6 ng/mL. Contrast-enhanced CT of the abdomen done on day 6 reconfirmed the finding of acute pancreatitis along with cholelithiasis and bilateral nephrolithiasis. There were no supra-renal masses. Ultrasound of the neck was normal. 99mTc-sestamibi scan revealed a 1.0x0.9 cm tracer-avid lesion suggestive of left inferior parathyroid adenoma. A diagnosis of hypercalcemic crisis secondary to PHPT was made. Suspecting multiple endocrine neoplasia syndrome, relevant investigations revealed normal serum prolactin, normal serum insulin-like growth factor 1 (IGF-1) (age and pubertal status matched), normal sella (on CEMRI), and non-elevated 24-hours urinary metanephrine and nor-metanephrine. Sanger sequencing for the *MEN1* gene did not reveal any mutation. Work-up for secondary causes of hypertension including renal artery Doppler, plasma aldosterone concentration/plasma renin ratio, and urinalysis were unremarkable. Hence hypertension was attributed to PHPT. He underwent open surgical excision of the left parathyroid mass. The remaining three parathyroid glands were explored and, in view of normal morphology, they were left *in situ*. Histopathology of the excised tissue showed parathyroid adenoma. Post-operatively his calcium and iPTH levels came down to 8.8 mg/dL and 28 pg/mL, respectively. His sensorium completely improved on day 1 post-surgery. He was taken off antihypertensive medications and discharged on day 4 post-surgery. At follow-up, he remained normotensive and normocalcemic. MRI brain repeated after three months post-surgery showed complete resolution of prior changes.

Informed written consent was obtained from the patient’s father.

### Case 2

**Figure 2:**
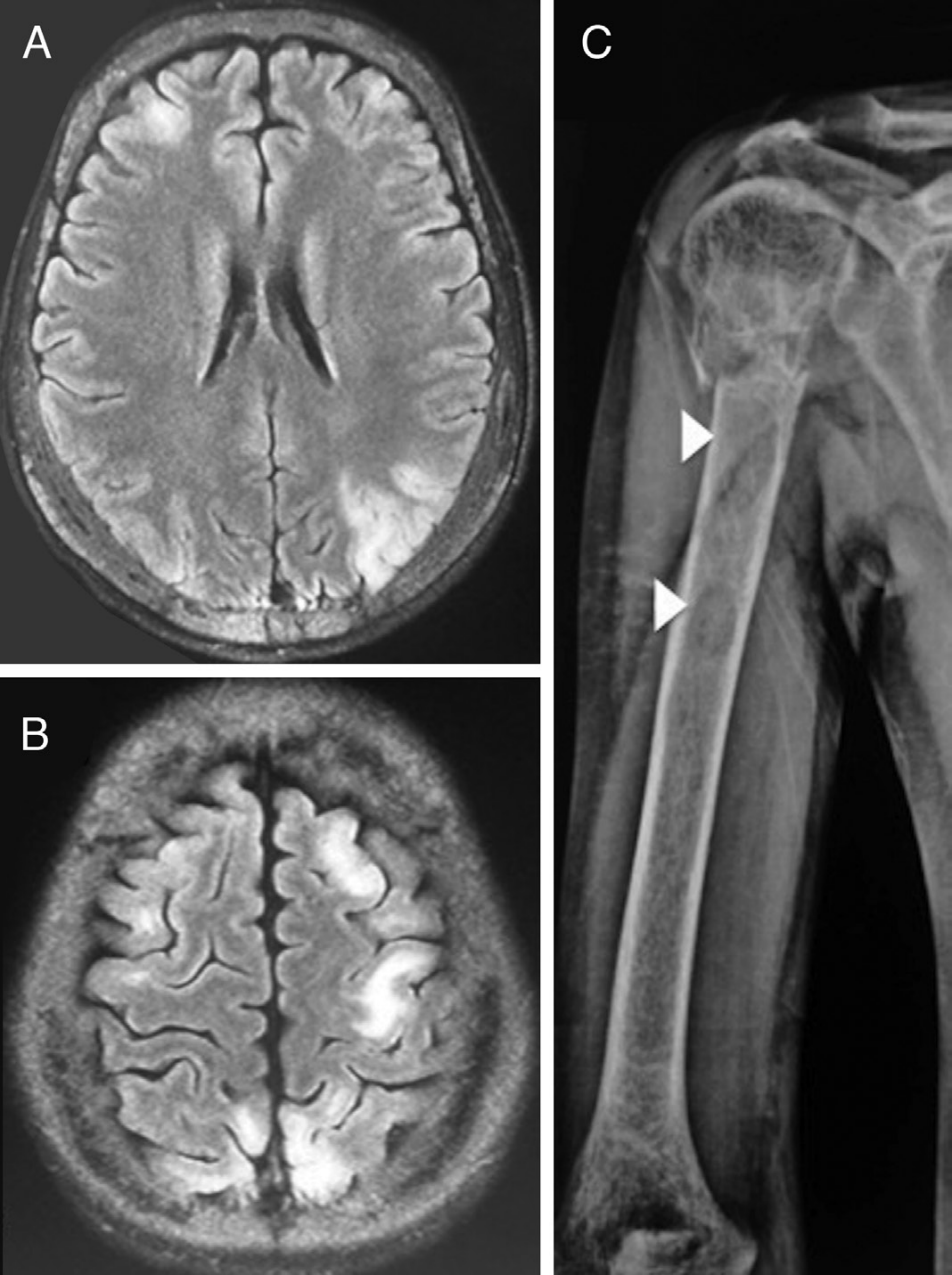
A, B) Magnetic resonance imaging of brain (second case) with T2/fluid-attenuated inversion recovery images showing hyperintensities in the parietal, occipital and frontal regions, more marked on the left side. C) Radiograph of the right shoulder and arm showing a displaced fracture of the surgical neck of the right humerus. Two lytic lesions are seen in the shaft of the right humerus suggestive of brown tumors of hyperparathyroidism (marked in white arrow heads)

A 16-year-old boy presented with a two day history of altered sensorium following an episode of GTCS. His parents had noticed an alteration in his behavior over the past two weeks, in the form of apathy, irritability, and decreased alertness. Past medical history was relevant in that he had suffered fractures of his right humerus and right neck of femur following a fall from a motorcycle one month previously and was immobilized in plaster casts at a local hospital. At presentation to the ED, he had altered mentation with a GCS of 11/15 (E_3_V_3_M_5_). The right lateral margin of his tongue was lacerated suggestive of tongue-bite. He was found to have hypertension (BP=180/100 mmHg). The fundus examination was unremarkable with no evidence of papilledema. Neck rigidity was absent. Preliminary investigations revealed hypercalcemia (corrected serum calcium 14.5 mg/dL), hypophosphatemia (serum phosphate 1.1 mg/dL), normonatremia, normokalemia and normal renal function. The cerebrospinal fluid analysis was normal. Non-contrast CT of the head showed multiple lytic lesions in the calvarium, while brain parenchyma appeared grossly normal. Hence, a CEMRI brain was performed which showed areas of cortical and subcortical white matter hyperintensities on T2/FLAIR-weighted images with diffusion restriction involving the parietal, occipital, and frontal regions (predominantly left-sided), suggestive of PRES (Figure 2A, 2B). Hypercalcemia was managed with parenteral fluids and parenteral zoledronic acid while hypertension control required labetalol infusion. Detailed workup for the cause of hypercalcemia revealed iPTH of 2491 pg/mL and 25-hydroxyvitamin D of 18.38 ng/mL. Ultrasonography of the neck was non-contributory, however, 99mTc-sestamibi scan revealed a 2.5x1.6 cm left inferior parathyroid adenoma. Radiographs showed fractures of the neck of right femur and surgical neck of the right humerus, multiple lytic lesions (suggestive of brown tumors) (Figure 2C), diffuse cortical thinning of long bones, and sub-periosteal resorption of the phalanges of fingers. Dual-energy X-ray absorptiometry was suggestive of low bone mineral density. Abdominal ultrasonography revealed a small right renal calculus. Secondary causes of hypertension, such as renal parenchymal disease, renal artery stenosis, pheochromocytoma, primary aldosteronism, Cushing’s syndrome, and hyperthyroidism were diligently ruled out. Hence, a clinical diagnosis of PHPT was made; hypertension and PRES were attributed to the hypercalcemic crisis. In view of severe hypercalcemia, iPTH level more than 10 times upper limit of normal, young age, male gender, concomitant bone, and renal involvement, a possibility of parathyroid carcinoma was considered (16,17,18). Lack of similar family history, normal serum prolactin, and age/pubertal status matched serum IGF-1 levels, normal sella (on CEMRI), absence of any thyroid nodule and non-visualization of any jaw lesion on imaging made syndromic causes of PHPT less likely. Sanger sequencing for the *MEN1* gene did not reveal any mutation. By day 3 of admission, his serum calcium came down to 12.3 mg/dL and there was a marked improvement in his sensorium. On day 5 he underwent excision of the left inferior parathyroid mass; the mass was not infiltrating the surrounding tissues and could easily be dissected out. The remaining three parathyroid glands were explored, however, they appeared morphologically normal to the operating surgeon. His calcium and iPTH levels came down to 9.4 mg/dL and 43 pg/mL, respectively on day 1 post-surgery. Histopathology of the excised lesion was suggestive of parathyroid adenoma with no features of parathyroid carcinoma. His sensorium completely improved; his anti-hypertensive requirement came down and he was discharged on 5 mg of amlodipine. At one month follow-up, he was normocalcemic. His BP was in the low-normal range; hence, amlodipine was stopped. When reviewed at three months, he was normotensive and normocalcemic. A repeat MRI brain showed a complete resolution of the T2/FLAIR hyperintensities.

Informed written consent was obtained from the patient’s father.

## Discussion

Herein we have reported two cases of PHPT presenting with predominantly neurological complaints and diagnosed as having PRES. Both of them had hypertension and severe hypercalcemia (serum calcium >14 mg/dL) at presentation. Neurological manifestations, hypertension, and MRI changes resolved following parathyroid adenomectomy and restoration of normocalcemia. These two cases add to the small list of reports of PHPT presenting as PRES. These cases are however very unusual as both of them were young. PHPT *per se* is an uncommon endocrine disease in the pediatric population with a prevalence of 2-3 cases per 100,000 (19). Similarly, PRES in the pediatric/adolescent population is even less common with mostly anecdotal case reports and a few small case series (20,21). No case of PRES in young PHPT has hitherto been reported.

PRES, also known as reversible posterior leukoencephalopathy syndrome, is a clinico-radiological entity characterized by acute neurological symptoms. First described in 1996, the entity remains rare with its global incidence being unknown. Most cases occur in young-to-middle aged adults with a female preponderance. PRES is commonly seen in the setting of renal failure, pre-eclampsia/eclampsia, and accelerated hypertension. Acute hypertension, more precisely abrupt fluctuations in BP cause endothelial dysfunction, breakdown of the blood-brain barrier, and subsequently vasogenic edema, leading to PRES (1). The predominant involvement of the posterior regions of the brain in PRES is primarily believed to be due to lower density of sympathetic innervation of the vertebrobasilar system, a factor that maintains cerebral autoregulation and protects the brain from severe hypertension (22). Hypertension in PRES is however not universal; 15-20% of patients are normotensive or even hypotensive (23). Endothelial dysfunction and subsequently interstitial brain edema in such cases is mediated by excessive circulating cytokines. PRES occurring in the setting of underlying autoimmune diseases, post-organ transplant, cytotoxic/immunosuppressive drug use, and sepsis appears to be mediated predominantly by cytokines (1).

Hypercalcemia is rarely cited as a cause of PRES. Multiple mechanisms have been proposed for hypercalcemia-induced PRES. Vasospasm of the cerebral vessels being one of them (5,8). Hypercalcemia leads to augmented actin-myosin coupling, resulting in vascular smooth muscle contraction and subsequent vasospasm in the cerebral circulation (8). The subsequent perturbations in cerebral blood flow lead to endothelial cell injury, culminating in PRES (6). In addition, a sudden rise in BP induced by acute hypercalcemia can precipitate PRES. Hypertension in such settings is mediated not only by a direct effect of calcium on vascular smooth muscle but also by an indirect effect of calcium-mediated hypercatecholaminemia (24). High levels of circulating calcium can directly lead to endothelial dysfunction. Rats with diet-induced hypercalcemia exhibit a transformation of their endothelial cells to a predominantly pro-inflammatory phenotype (25). Hypercalcemia has been shown to increase the expression of renal endothelin-1, inducible nitric oxide synthase, and other pro-inflammatory cytokines in rats (26,27). Lastly, hypercalcemia-induced hypomagnesemia has been proposed as one of the underlying mechanisms in the failure of cerebral autoregulation (3). Thus, cerebral vasospasm, acute hypertension, endothelial dysfunction, and hypomagnesemia provide an optimum milieu for precipitating PRES.

Amidst the plethora of patients with hypercalcemia encountered in routine clinical practice, the rarity of occurrence of PRES needs to be explained. The extent of hypercalcemia perhaps dictates the pathogenesis of PRES. In all the hitherto reported cases of hypercalcemia and PRES, the corrected serum calcium levels were more than 13 mg/dL (3,4,5,6,7,8,9,10,11,12,13,14,15). Accordingly, hypercalcemia-induced PRES has mostly been recognized either in the setting of malignancy or iatrogenic vitamin D/calcium overdose (3,4,5,6,7,8,9,10). PRES in the setting of PHPT is extremely rare. This probably reflects the relatively mild-moderate levels of serum calcium seen in PHPT patients. In a registry of 464 patients with histologically proven PHPT, the mean calcium level was 11.9 mg/dL (28). In addition to absolute calcium levels, the rate of rise in serum calcium is probably equally important. Hypercalcemia is of more rapid onset in malignancy when compared to PHPT (29). The relatively rapid rise in serum calcium in association with underlying malignancies perhaps causes sudden perturbations in cerebral blood flow, leading to PRES. Alternatively, the rarity of association can also be explained on the basis that most patients with hypercalcemia presenting with altered sensorium do not undergo neuro-imaging, thereby leading to under-diagnosis of PRES.

After an extensive literature search, thee were only four cases of PHPT associated with PRES (Table 1) (12,13,14,15). The case reported by Popkirov et al (15) cannot strictly be labeled as PHPT; rather it was a case of tertiary hyperparathyroidism developing in a patient with hereditary hypophosphatemic rickets following long-term phosphate supplementation. All but one patient was male; all were above 50 years of age. The predominant clinical manifestations were seizures and altered sensorium; other associated symptoms included headache, visual hallucinations, visual field defects, aggressive outbursts (12) and worsening of extrapyramidal disease (14). Hypertension at presentation was seen in all but one patient. All had severe hypercalcemia, ranging from 14.3 mg/dL to 21.2 mg/dL. Consistent with the diagnosis, all had elevated serum PTH levels. A parathyroid adenoma was located in all four cases. Parathyroid adenomectomy led to normalization of serum calcium along with a rapid and complete resolution of the neurological symptoms. The case reported by Au et al (13) however had residual neurological deficits in the form of persistent left-sided homonymous hemianopsia and neglect, likely because of underlying hypoxic brain damage as the patient had been in status epilepticus for six days. The resolution of MRI changes had been documented in only two cases with normalization occurring as early as seven days following the restoration of normocalcemia. Okaygün et al (30) reported a case of PHPT with severe hypercalcemia, pancreatitis, and encephalopathy; cranial CT revealed periventricular ischemia, however, the diagnosis of PRES is debatable.

The sequence of events leading to PRES in our two cases needs clear explanation. Severe hypercalcemia (serum calcium >14 mg/dL) had precipitated the hypercalcemic crisis. However, as has already been said, the occurrence of severe hypercalcemia in PHPT is a rarity; only 6% of PHPT patients treated at the Surgical Service at the University of Michigan Hospital during a 16-year period had severe hypercalcemia. Hypercalcemia would have subsequently led to hypertension, although, high BP has been recorded in normocalcemic PHPT as well. Higher levels of pressor hormones and increased cardiovascular reactivity to catecholamines have been implicated as the cause of hypertension in PHPT. Hypercalcemia and hypertension would have subsequently worked in tandem to precipitate PRES. Acute pancreatitis, as present in our first case, might also have contributed to the occurrence of PRES. Hypercalcemia in the second case might have been further aggravated following immobilization after sustaining a fracture of the right neck of femur. Besides, PTH *per se* has been implicated in directly causing endothelial dysfunction. Although PTH-induced endothelial dysfunction can lead to PRES in PHPT, the same does not hold true in PTH-independent causes of hypercalcemia as PTH levels are suppressed. This implies that hypercalcemia is more important in the causation of PRES than elevated PTH levels.

## Conclusion

In conclusion, we have presented two cases of young PHPT presenting as PRES. Severe hypercalcemia and hypertension were common to both; MRI was suggestive of T2/FLAIR hyperintensities predominantly affecting the occipito-parietal regions. Neurological symptoms and MRI changes resolved with the restoration of normocalcemia following parathyroid adenomectomy. We therefore propose that serum calcium levels should be checked in all patients with PRES and that PHPT be considered as a differential diagnosis in those with underlying hypercalcemia.

## Figures and Tables

**Table 1:**
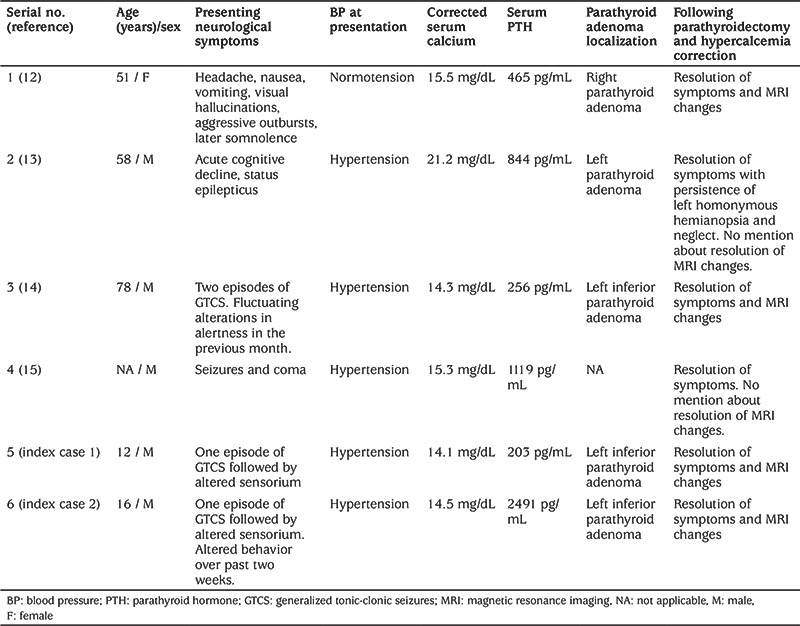
Four cases of primary hyperparathyroidism-associated posterior reversible encephalopathy syndrome hitherto reported in the world literature. The two index cases described herein have also been included in the table
